# Photocatalytic degradation of aquatic organic pollutants with Zn- and Zr-based metal-organic frameworks: ZIF-8 and UiO-66

**DOI:** 10.55730/1300-0527.3444

**Published:** 2022-08-11

**Authors:** Fatma Defne ÇALIK, Bilgesu ERDOĞAN, Esra YILMAZ, Gizem SAYGI, Fehime ÇAKICIOĞLU-ÖZKAN

**Affiliations:** Department of Chemical Engineering, Faculty of Engineering, İzmir Institute of Technology, İzmir, Turkey

**Keywords:** Photocatalysis, photocatalytic reaction mechanism, metal-organic frameworks, UiO-66 and ZIF-8, wastewater treatment, organic contaminants

## Abstract

Water treatment has been an essential issue with the increasing population over 40 years. Researchers center on the major organic pollutants, such as dyes, pesticides, and pharmaceutical products. Photocatalytic degradation is one of the promising methods for aquatic organic pollutant treatment. Over the years, scientists have been working on developments for photocatalysts to enhance their pollutant degradation performances. From the reviewed studies, it is seen that properties like surface area, chemical, mechanical, and thermal stability, and uniform distribution of active sites are crucial, and an increase in these properties provides better degradation efficiency. In this sense, metal-organic frameworks as photocatalysts can be considered more advantageous. This study focuses on the organic aquatic pollutant degradation studies by using well-known MOFs like ZIF-8 and UiO-66 photocatalysts. Mainly the organic dye (RhB, MB, MO, etc.) degradation efficiencies of ZIF-8 and UiO-66 have been achieved to 100%. Recently, the degradation capacities of various pharmaceuticals such as diazinon, acetaminophen, levofloxacin, and sulfamethoxazole have also been investigated. According to the reviewed studies, ZIF-8 and UiO-66 can be considered remarkable photocatalysts for the degradation of organic pollutants.

## 1. Introduction

Due to industrialization and improvement in living conditions, an increase in the human population has been observed [1]. However, recent population and developed industrialization have caused severe damage to the ecosystem [2]. Considering the world’s water resources, while 80% of the world is covered with water, 97% of it originates from oceans and seas. Due to its salinity, water from oceans and seas is not available for human usage. Tons of water is polluted every day by industrial and household wastes, fertilizers, and drugs used in agriculture. When this polluted water is removed from the diagram, only one-third of the potential freshwater is suitable for human use [3]. According to the World Health Organization, 35% of the world’s population does not have access to improved sanitation [4]. More than 3.4 million people, primarily children, die in a year due to water pollution [5].

There are many different contaminants in water from different sources. These contaminants are divided into two main branches: organic and inorganic contaminants. Apart from these two, pathogens, radionuclides, and macroscopic contaminants are also present in water. Inorganic pollutants are more permanent in the environment than organic ones. They can dissolve in water and are very active in water [6]. They can accumulate in the human body and the food chain. This accumulation of pollution has toxic and even lethal effects on living beings.

Organic pollutants, on the other hand, have more variety than inorganic ones. Although they are less permanent than inorganic pollutants, they have become more permanent in modern society due to their continuous use [6]. For this reason, studies have been carried out to remove organic pollutants from nature by many different branches from past to present.

Numerous investigations have been reported for removing organic pollutants from wastewater, including chemical precipitation [7], coagulation [8], filtration [9], photocatalysis [10,11], and adsorption [12]. Among them, photocatalysis has significant advantages, including ambient operating temperatures, low operating cost, and complete mineralization of organic pollutants to CO_2_ and H_2_O. Photocatalysis is reaction series induced by visible or invisible light and accelerated by a catalyst called photocatalyst. A photon initiates these reaction series with the proper energy equal to or higher than the photocatalyst’s band gap energy. The commonly used semiconductor (photocatalyst) materials are TiO_2_, ZnO, Fe_2_O_3_, Bi_2_WO_6_, and C_3_N_4_ [13]. As photocatalysts, metal-organic frameworks (MOFs) like UiO-66 and ZIF-8 materials also gain attention. These MOFs are promising due to their reusability, high surface area, eco-friendliness, stability, and activity.

This research summarizes the organic aquatic pollutants, their effects on the environment, removal methods, and the degradation of these pollutants by using MOF-based photocatalysts such as ZIF-8 and UiO-66.

## 2. Organic aquatic pollutants

### 2.1. Sources, types, and characteristics of aquatic pollutants

Organic components have more variety and branching in nature than inorganics. For this reason, organics appear as contaminants more than inorganics. The studies on pollutants have also intensified due to the recently increasing pollution in water resources [6]. Organic pollutants in water can be examined in many subtitles. However, it is proper to examine them in 5 main branches: agricultural products, dyes, pharmaceuticals and personal care products, artificial sweeteners and food additives, and plastic products.

#### 2.1.1. Agricultural products

The rapid increase in the human population creates more need for agricultural goods. The scarcity of lands to be planted and the need for more agricultural products increase the use of pesticides [14]. Insecticides and growth promoters are the two most significant sources of agricultural pollutants. Not only do they pollute the soil, but they also mix with groundwater and spread to the entire ecosystem [15]. More products can be produced using pesticides, negatively affecting the world health [16]. Pesticides can be classified as insecticides, herbicides (for weeds), rodenticides (for rodents), fungicides, acaricides and miticides (for ticks and mites), molluscicides (for mollusks), bactericides (for bacteria), avicides (for bird pests), virucides (for virus), algicides (for algae) [16,17]. Almost all pesticides have toxic effect, and they are also stable in the aquatic environment [17].

#### 2.1.2. Dyes

Synthetic dyes are widely used in many industries such as textile, printing, cosmetics, food, papermaking industry, and medicine [17,18]. Dyes are classified as azoic, reactive, vat, sulfur, acid, disperse, direct, and basic [19]. Among these species, azo-dyes used in almost every industry are the most polluting types [18–20]. However, azo-dyes with nitrogen bonds in their structure can also be used in antibacterial areas [20]. The food industry is another area where azo-dyes are frequently used. The paint in foods offered in different colors to draw people’s attention is kept under control with certain restrictions [18]. Despite the contributions from all these different industries, the textile industry is leading to water pollution. It is necessary to wash the dyed or printed fabric and thread with water to make it more useful in the textile industry. Caustic soda-based soaps, enzymes, and various solvents are often added to this washing water [21,22].

#### 2.1.3. Pharmaceuticals and personal care products

Pharmaceuticals are a group of chemicals used to diagnose, treat, and prevent diseases used to treat humans and animals. Many drugs, including antibiotics, synthetic hormones, statins, and cytotoxins, are consumed by humans. These drugs, in large quantities, interfere with urban wastewater [23,24]. Antibiotics are used in agriculture and aquaculture to prevent disease and promote growth. In humans and animals, 30% to 90% of antibiotics are thought to come out unchanged from the body. [25]. This unchanged content of consumed antibiotics is mixed with wastewater. The mixing of antibiotics with water causes a danger to human health. In addition, the excessive and incorrect use of antibiotics increases antibacterial resistance. Use of cosmetics, perfumes, and personal care products increases as well-being increases. The recent increase in the usage rates of these products has caused these products to be added to water sources as pollutants [26].

#### 2.1.4. Artificial sweeteners and food additives

Sweeteners are additives used to sweeten foods by keeping them low in calories. In addition to natural sweeteners from sugarcane and sugar beet, artificial sweeteners are also used in the food industry. Examples of common artificial sweeteners include saccharin, aspartame, acesulfame, cyclamate, and neotame. They are actively used in many areas, from jams to beverages in the food industry [27,28]. Acesulfame K is the compound with the highest pollution rate among these additives. It is preferred due to being 200 times sweeter than sucrose and is used in many areas, such as canned foods, cereals, confectionery products, and tabletop sweeteners. In addition to being used alone, it is used synergistically with other sweeteners to increase flavor. The main problem with these synthetic sweeteners is that they leave the body unchanged due to the lack of metabolism in the human body. The amount of sweetener observed in surface waters is higher than the average pollutant concentration. Despite scientists’ search for ways to inactivate it, the desired results could not be obtained precisely because the residue produced from inactivation had a more toxic property than the pollutant itself [29]. Food additives are used for many different purposes, not just for sweetening. In order to increase the quality, sensory properties, and shelf life of food and to maintain its nutritional value and safety, different additives are used to facilitate processing, storing, and transporting food and meet the expectations of customers’ needs [30].

#### 2.1.5. Plastic products

The increase in the production trend of plastic, one of the most popular materials of our time, has reached such dimensions that cannot be ignored from year to year. Global production, which was 50 Mt/year in the 1970s, reached almost 300 Mt/year in 2015. Worm et al. argued that of the plastics production in 2015, 40% was used in packaging, 20% in consumer items, 20% in construction products, while the remaining 20% was used in most automotive, electrical, and agriculture applications [31]. Since their recycling and usage are not done correctly, it poses a significant threat to the world and the ecosystem. Approximately 70% of the garbage existing in the world consists of plastics. It is not easy for plastic to disappear spontaneously in nature, and almost 10% of these plastics produced are found as pollutants in the seas. It is possible to list the entry points to the sea as beaches, rivers, land waste transport by wind, and wastewater discharges. Apart from these land-based pollutants; maritime activities, fishing gear, and some shipwrecks are also among the reasons plastics are included in water sources. Each plastic mixed with these resources will cause economic damage to the marine economy, but its consequences will be serious in terms of biological and ecological meanings [32].

The main additives commonly used in plastics, such as phthalates, bisphenol A (BPA), alkylphenols, and polybrominated diphenyl ethers, are very harmful to humans’ biology [32,33]. These are classified as endocrine-disrupting chemicals (EDC) and cause changes in hormone balances in the human body. Moreover, plastics are used today as raw, main, intermediate, and additives [31]. Thus, all these compounds have been measured in large quantities, even in open seas, especially on beaches. In addition to water pollution, dioxins, furans, mercury, and polychlorinated biphenyls are highly toxic gases. Due to the incineration of plastics during their conflict, these gases mix with the air [34].

### 2.2. Health and environmental effects of organic aquatic pollutants

Pesticides are non-biodegradable molecules. If possible, these molecules should not be allowed to mix into the natural environment. Pesticides are a type of pollutant that can harm people through water and the air. Exposure to these pollutants can lead to many adverse conditions such as vector-borne diseases, asthma, allergies, microbial contamination, avian influenza, prions, and anthrax [15,35].

Many of the colorants are classified as carcinogens. As the wastewater released in the textile industry meets freshwater, dyes and other effluents are included in the ecosystem. All these pollutants damage the creatures in the water before they reach human beings. These organic substances in textile wastewater react highly with disinfectant molecules. In this way, they enter the breathing air, are absorbed by the skin’s surface, and cause allergic reactions. This carcinogenic property generally results from the aromatic amine portions of azo-dyes. In addition to being used as a hair dye component, p-phenylenediamine, which is also used in engineering polymers and composites, has been shown to cause allergies, throat irritation, bronchial asthma, and sensitization dermatitis. In addition to causing severe eye and skin irritations, it has been noted that it can cause dizziness, drowsiness, shortness of breath, and serious liver damage [20].

According to the data obtained, personal care and pharmaceuticals are the products that cause the greatest harm to human metabolism and even deoxyribonucleic acid (DNA) of humankind. Since they are involved in human metabolism, they enter many systems and create irregularities in the operation of the systems. Although it is the cause of many diseases, especially diabetes, it has been observed to leave the whole body vulnerable during pregnancy [36–38]. Antibiotics, which pass into the water at high rates due to combining household and hospital wastes by combining them in a shared pool, are another species that threaten human health and the ecosystem. The resistance of bacteria to antibiotics increases when these drugs and the water ecosystem are mixed. In this way, the human body becomes more vulnerable to bacteria [25].

All artificial sweeteners from households and industries are found in large quantities in wastewater and are difficult to separate. Combining with groundwater, they mix with drinking water and get the chance to reenter the human body [27,39]. In addition to human health, it causes disruptions and negativities in the life of aquatic creatures [40]. Even if the values in the food are kept under control, it is essential to test the amounts that accumulate in the waters after use. Although it is thought that it will not cause problems when taken into the body at low levels, the continuity of the intake and the reactions they will enter with other components are debatable [39,41]. Although it is used in the food industry because some reduce the risk of diabetes, there are reports that it causes seizures, shortness of breath, allergies, headaches, cancer, chromosomal changes, and even cancer [42–45].

Unfortunately, the journey of plastics mixed with water in different ways ends in the human body. BPA, polystyrene, polyethylene, polyester, and polyvinyl chloride are available in many materials such as phthalates water and damage the human body due to toxicity. The first prominent place people are exposed to plastic is the water sold in plastic bottles. Reinforcing substances added to the carton bottles to be durable also contain plastic. Significant amounts of microplastics were found in the water contents stored in glass bottles, which are the safest [46]. Plastic causes developmental disorders in children, diseases caused by EDCs, and congenital disabilities. The harm of plastics to the human body is as severe as lead, cadmium, and mercury. A summary of the health effects of the contaminants is given in Table 1.

### 2.3. Wastewater treatment methods

The types and dosages of pollutants in drinking water are of great importance. While some pollutants in small amounts are not a big problem, in some cases, even the smallest doses create severe health and environmental problems [27,44]. The maximum contaminant level goal (MCLG) is the level of pollution that will not pose any known risk to health. The maximum contaminant level (MCL) is the amount of contaminant in the water without harming the human body. Appropriate treatment techniques (TT) should be used when this amount is exceeded. These doses are specified in the United States Environmental Protection Agency’s standards, and values for some pollutants are listed in Table 2.

In addition to actively installed facilities to clean contaminated water, efforts are underway to make cleaning better and more cost-effective. It is possible to examine wastewater treatments in 3 main branches: Physical, chemical, and biological methods.

Using only one of these processes does not purify wastewater completely. Because generally, wastewater does not contain only one type of pollutant. For example, real textile wastewater contains dye, heavy metals, surfactants, solids, and various compounds [47]. By arranging all these methods in a specific frame, complete cleaning can be performed.

**Figure 1:**
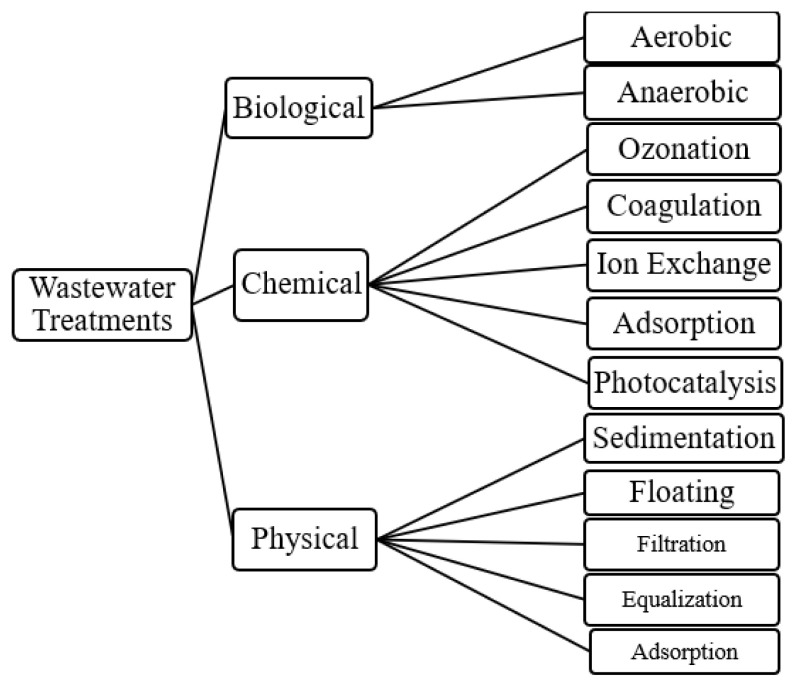
Wastewater treatments.

In addition to the method specified in Figure 1, new cleaning methods are also used [48]. Water cleaning studies using membrane technologies are tried for many pollutants [49]. Granular activated carbon is another widely used water cleaning method [50]. Another cleaning method that has come to the fore recently is photocatalysis. Photocatalysis removes the contaminants on the material’s surface under UV rays or sunlight.

## 3. Photodegradation of aquatic pollutants

Photocatalysis is one of the most promising methods for aquatic pollutant degradation with an unlimited energy source since it is initiated with photons with the proper energy. It is an advanced oxidation process where semiconductors are used to start a degradation reaction. Commonly known semiconducting materials are TiO_2_, ZnO, and Fe_2_O_3_. Photocatalysts can be modified by controlling morphology, synthesizing composites, and doping to enhance photocatalytic activity [51–53].

### 3.1. The basic mechanism of photocatalytic processes

Photocatalysis is a chemical reaction caused by photons that break down inorganic or organic chemicals. Reactions do not have to be carried out under visible light. Any photon with enough energy can create a transformation in the chemical bonds. Thus, X-rays, ultraviolet light, and gamma rays are some examples that can start photocatalysis reactions [54].

**Figure 2:**
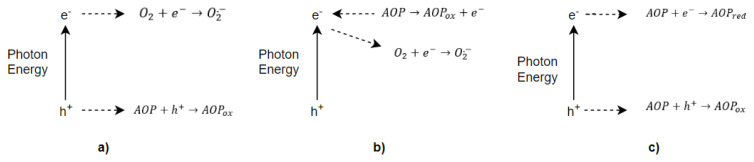
Photodegradation reactions for aquatic organic pollutants (AOP) a) oxidation reactions, b) electron injection, c) redox reactions.

The photocatalysis reactions start when a photon stimulates the photocatalyst with an energy higher than or equal to the band gap energy of the photocatalyst. The smallest energy difference between the conduction and valance band is band gap energy. Three reaction steps occur in the photodegradation process: oxidation (Figure 2a), electron injection (Figure 2b), and redox reactions (Figure 2c).

Upon irradiation, an electron transfer from the valance band to the conduction band starts (Eq. (1)). The excited electrons (e^−^) which left the valance band cause holes (h^+^) in the valance band. The photo-generated electrons possibly couple with the holes and release heat, as seen in (Eq. (2)).


(1) $$photocatalyst+h\upsilon \to e_{cb}^{-}+h_{vb}^{+}$$

(2) $$e_{cb}^{-}+h_{vb}^{+}\to heat$$

**Figure 3:**
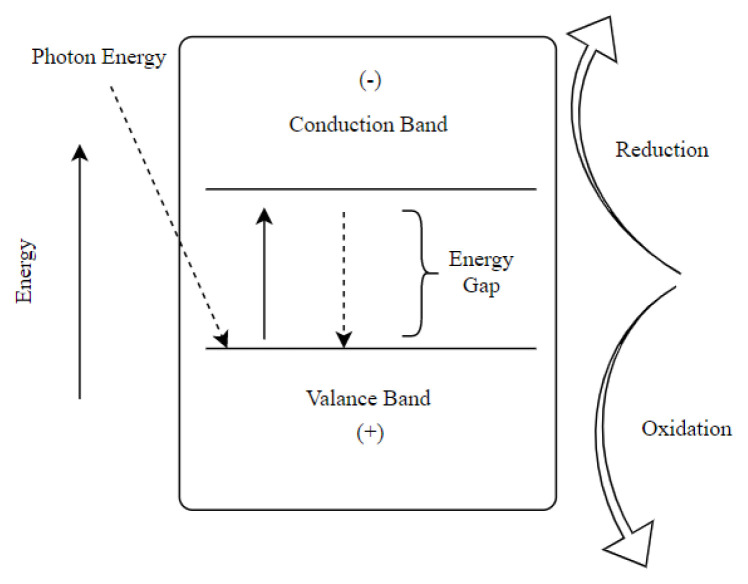
Mechanism of photocatalysis.

If enough time is supplied to the electrons and holes before coupling, they can move to the surface of the photocatalyst. These electron-hole (−) pairs are captured on the photocatalyst surface then undergo the redox reactions (Figure 3).

The positive holes in the valance band react with organic compounds, oxidize them, and lead to CO_2_ and H_2_O production. Another oxidation reaction is likely to occur when H_2_O and positive holes react to produce highly reactive hydroxyl radical (OH). This radical can oxidize almost all electron-rich organic molecules and produces H_2_O and CO_2_ [55]. These reactions can be seen in (Eqs. (3)–(5)).


(3) $$h_{vb}^{+}+organic\;compounds\to intermediates\to {CO}_{2}+H_{2}O$$

(4) $$h_{vb}^{+}+H_{2}O\to •OH+H^{+}$$

(5) $$•OH+organic\;compounts\to {CO}_{2}+H_{2}O$$

The electrons that move to the conduction band also react to prevent the accumulation of excess charges. Thus, the electrons react with the oxygen, creating superoxide anions, and the further reaction produces hydrogen peroxide (H_2_O_2_) and consequently hydroxyl radicals. Considering their oxidation potential (2.8 V), mainly the hydroxyl radicals (OH) react with the organic compounds and completely mineralize them in a nonselective way (Eqs. (6)–(11)) [56].


(6) $$O_{2}+e_{cb}^{-}\to O_{2}^{•-}$$

(7) $$O_{2}^{•-}+H^{+}\to HO_{2}^{•}$$

(8) $$2HO_{2}^{•}\to H_{2}O_{2}$$

(9) $$H_{2}O_{2}+h\upsilon \to •OH$$

(10) $$O_{2}+O_{2}^{-}\to •OH+{\;}^{-}OH+O_{2}$$

(11) $$H_{2}O_{2}+e_{cb}^{-}\to •OH+{\;}^{-}OH$$

**Figure 4:**
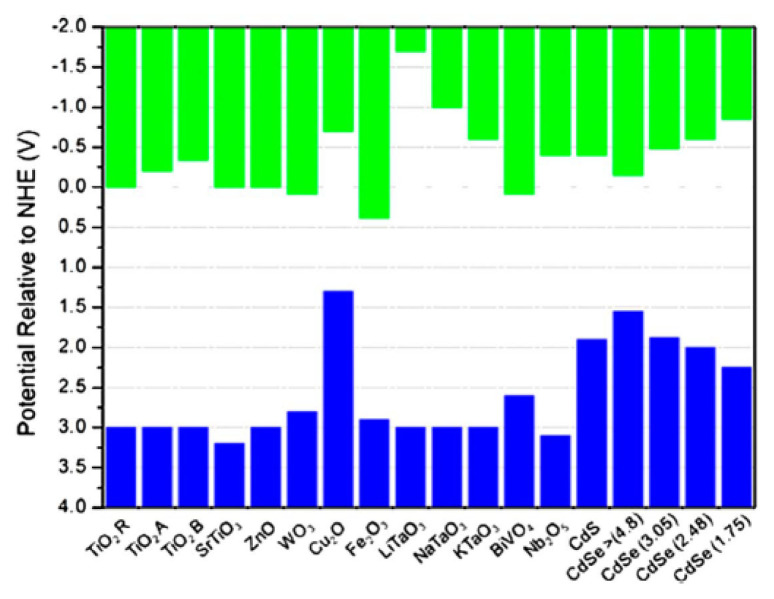
Band gap energies of different photocatalysts where blue columns represent the valance band and green columns represent the conduction band. Reproduced from ref. [56] with permission from the Royal Society of Chemistry.

Band gap energies of the semiconductors play an essential role in photocatalysis with respect to light absorption and converting the energy taken from the light into chemical energy to be used in the oxidation and reduction half-reactions. Band gap energies of the semiconductors ensure the feasibility of the proceeding reactions. Figure 4 shows the valance and conduction band potentials of the different photocatalysts. While developing a photocatalyst according to their application, it is necessary to consider the band gap energy, and band gap levels are arranged by controlling the particle size of the semiconductor [56,57].

### 3.2. Catalysts for the degradation of aquatic pollutants

The most common semiconductor photocatalysts used for water pollutant degradation are nonmetals, metals, and metal oxides like TiO_2_, ZnO, Fe_2_O_3_, WO_3_, BiVO_4_, and ZrO_2,_ as well as metal-organic frameworks like MOF-199, MIL-53, ZIF-8, and UiO-66. Scientists have worked on photocatalysts to enhance their degradation performances and developments like metal ion doping, composite photocatalysts, morphology control, nonmetal doping, and semiconductor heterojunction. These photocatalysts can be used to degrade aquatic organic pollutants such as dyes, pesticides, phenols, antibiotics, etc. [58]. TiO_2_ is a photocatalyst with low cost, nontoxicity, and availability [59]. However, its wide band gap causes a decrease in its effectiveness for using solar irradiation, and researchers are trying to overcome this inefficiency. Both Hu et al. [60] and Naik et al. [61] applied morphological change development to increase the efficiency of TiO_2_. Hu et al. [60] prepared a core-shell structured TiO_2_ with a hydrogen treatment method to degrade methylene blue. Naik et al. [61] prepared a super porous TiO_2_ with a basic sol–gel-assisted reflux method to degrade Amaranth dye. Consequently, in both studies, regarding the photocatalysis efficiency on the degradation of azo-dyes, morphologically changed TiO_2_ resulted better than the pristine TiO_2_.

ZnO has many attractive features like having exceptional electrochemical and optical properties and low cost and availability. It is possible to obtain different nanostructures of ZnO due to its versatility. Serrano-Lázaro et al. [62] established a flower-like nanostructured ZnO film with a spray hydrolysis method to degrade the widely used pesticide temephos in water. The efficiency of the ZnO photocatalyst increases due to the defects in ZnO lattice and flower-like morphology gained with nanostructural enhancement. Results show that nanostructured ZnO films are suitable for degrading temephos pesticides in water [62]. Micheal et al. [63] developed carbon nanoplate supported ZnO nanorods with a wet chemical process to degrade methylene blue dye. Compared to pristine ZnO, carbon nanoplate supported ZnO nanorods improve photocatalyst efficiency [63].

Research on bismuth as a photocatalyst has considerably increased in the last decade [58]. Both Zhang et al. [64] and Heidari et al. [65] studied the bismuth-based photocatalysts and used the heterojunction to enhance the photocatalytic activity. The heterojunction is a semiconductor modification that contains a pair of two different semiconductor materials [66]. Zhang et al. [64] produce a 2D/3D Bi_5_O_7_Br/BiOBr heterojunction photocatalyst with a facile hydrolysis process to degrade carbamazepine drug in water. The results show that Bi_5_O_7_Br/BiOBr heterojunction shows a better photocatalytic performance than Bi_5_O_7_Br and BiOBr separately. Heidari et al. [65] also prepare BiOBr heterojunction photocatalyst, a 3D-flower-like BiOCl/BiOBr-Bi_24_O_31_Br_10_ type-II nano heterojunctions with the sono-assisted solvothermal method to degrade the four different types of fluoroquinolones such as levofloxacin, ofloxacin, norfloxacin, and ciprofloxacin. Similarly, Zhang et al. [64] also show that the nanoheterojunctions provide better performance than the pure component.

Researchers also use MOFs as semiconductors to degrade organic pollutants [64,67] by photocatalysis. MOFs are generally more advantageous for their greater internal surface area and easily adjustable organic units than common semiconductors [68]. Li et al. [69] and Fakhri and Bagheri [67] worked with MOFs to degrade pollutants in water bodies under visible light irradiation. In the study by Fakhri and Bagheri [67], UiO-66@ WO_3_/graphene oxide (UiO-66@WG), which is a MOF-based nanocomposite, was produced with the solvothermal method and studied in the degradation of tetracycline and malathion. Similarly, Li et al. [69] investigated the degradation of tetracycline and carbamazepine, bisphenol, and p-nitrophenol phenolic micropollutants. They prepared the g-C3N4/PDI@MOF heterojunction with the in situ growth of NH_2_-MIL-53(Fe) onto the g-C_3_N_4_/PDI layer. Considering the mutual pollutant tetracycline, the study by Li et al. [69] resulted in a better performance with 90% efficiency in 1 h, while the efficiency obtained by Fakhri and Bagheri [67] was 84% in 70 min. In addition, both studies showed superior performances compared to their respective pristine semiconductors.

### 3.3. Key factors affecting photocatalysis

Contaminant concentration, pH, and catalyst loading are the key factors for the organic contaminant degradation with photocatalysis.

Naik et al. [70] investigated the effect of catalyst and pollutant concentrations and pH on the degradation efficiency of Amaranth azo-dye with mesoporous anatase TiO_2_. It was observed that with increasing the catalyst amount to 110 mg, the degradation efficiency increased due to the increase in the surface area for adsorption. The efficiency observed at the optimum catalyst amount was 99.1% degradation of Amaranth azo-dye in 60 min. Later, the effect of pollutant concentration was evaluated. At the lower concentrations of the pollutant, the degradation occurred faster. Increasing dye concentration blocked the light photons from reaching the catalyst surface and thus decreased the degradation efficiency significantly. Finally, they studied the effect of the pH of the pollutant solution, and the most effective degradation was observed at the pH of 2 with 97.14% [70].

Yusuff et al. [71] studied the degradation of organic textile contaminants in water with ZnO/pumice photocatalyst. Their results show that the increased pH values cause a slight decrease in the efficiency, which indicates that the dye effluent is acidic [71].

In the research by Fakhri and Bagheri [67], the effect of composite dosage and pH on the degradation of aromatic tetracycline and nonaromatic malathion pollutants by UiO-66@WG photocatalyst was investigated. Different composite dosages were studied, and the best results were obtained with the 35% WO_3_/graphene oxide dosage for tetracycline and malathion. The best photocatalytic activity was found at a pH of 7 and 9, respectively, for the degradation of tetracycline and malathion [67].

Li et al. [69] investigated the most effective way for the degradation of pharmaceutical pollutants by observing the change of different catalyst dosages and H_2_O_2_ concentrations. An efficiency increment was observed when the catalyst dosages increased from 0.1 g/L to 0.2 g/L, but the efficiency remained constant after 0.2 g/L. The high amount of catalysis caused competing reactions; therefore, the photocatalytic efficiency was reduced. The optimum H_2_O_2_ concentration was determined as 10 mM for the degradation of bisphenol A and p-nitrophenol [69].

## 4. Effect of ZIF-8 and UiO-66 on the degradation of aquatic pollutants

In recent studies, it has been observed that MOFs are promising for breaking down aqueous organic pollutants with photocatalysts [72]. MOF is a porous material comprising inorganic metal ions and organic ligands. Some of the advantages of the photoactive MOFs are their well-defined crystalline structures, adjustable active sites, internal synthesis, and ability to design the photocatalysts properly [73]. The most common photocatalysts for the degradation of organic aquatic pollutants are graphene oxide or metal nanoparticles (i.e. Ag, Fe) supported or promoted MOFs, such as MILs, ZIF-8, and UIO-66 [73–75]. ZIF-8 and UiO-66 have mainly used photocatalysts for removing the aqueous organic pollutants. Deposition of metals (Ti, Ag, etc.) and/or immobilization of MOFs on the support surface have increased the photocatalytic activity [75,76].

### 4.1. Design of ZIF-8 and UiO-66 photocatalysts

Zeolitic imidazolate framework-8 (ZIF-8) consists of imidazolate organic ligands and Zn^+2^ metal ions. The higher thermal stability (up to 550 °C) and chemical stability (boiling alkaline water and organic solvents) puts it ahead of other MOFs. ZIF-8 structure can be obtained by many different synthesis methods: mechanochemical, solvothermal, sonochemical, microwave-assisted, dry gel, and microfluidic methods. Particle sizes, textural, surface, and structural properties, and production efficiency are directly dependent on the method used and the ambient conditions of the method [77]. To briefly explain some essential methods, one of them is the mechanochemical method that has been used recently due to the necessity for a more straightforward application and cheaper equipment.

Moreover, the chemical reactions during the grinding process can provide products without the need for high temperatures [78]. The mechanochemical method is a very efficient method thanks to the lower raw material loss, cost, and the resulting by-products for the production of ZIF-8 [79]. Solvothermal synthesis can be carried out with different solvents at certain temperatures. Furthermore, when the used solvent is water, which is called hydrothermal synthesis, is also a safer synthesis method for MOF production [80,81]. In sonochemical synthesis, an accelerated reaction takes place with the help of ultrasonic waves [82]. In some cases, it has become possible to harmonize the methods. For example, Nie et al. [83] used the solvothermal method in their study, benefited from ultrasonic waves’ properties, and named the method high efficiency.

**Figure 5:**
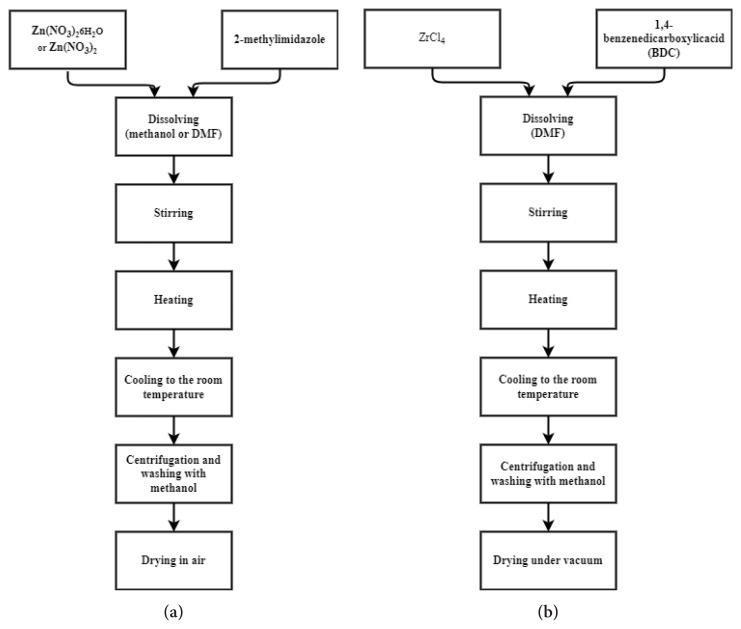
a) ZIF-8 synthesis, b) UiO-66 synthesis.

Besides, this eco-friendly photocatalyst can be produced easily in a short time, can be used again and again, and does not require spending lots of solvents during its production. In addition, the large surface area (BET specific surface area (SSA) (~2000 m^2^g^−1^), Langmuir surface area (~1810 m^2^g^−1^)) and desired pore size (pore cavities of ~1.16 nm and pore volume of ~0.60 cm^3^g^−1^) of ZIF-8 make it attractive for photocatalytic degradation of pollutants. As proposed by Chandra et al. [84], ZIF-8 can be obtained by using hydrated Zn(NO_3_)_2_ and 2-methylimidazole as dissolved in methanol [85,86] or DMF [87,88] and then stirred. The detailed procedure is given in Figure 5a.

One of the most attractive aspects of MOF synthesis is that the organic bridging ligand can be synthetically modified to introduce a desired functionality to the framework. The thermal stability of MOFs is generally 250–400 °C, and the chemical stability is quite low [89]. However, UiO-66 has great thermal stability until 540 °C and high chemical stability for water, acetone, benzene, and dimethylformamide. Cavka et al. [90] and Venna et al. [91] reported the synthesis procedure of a Zr-based MOF. ZrCl_4_ and 1,4-benzene dicarboxylate (BDC) were mixed with dimethylformamide (DMF) for a solvothermal process in an autoclave at 666 °C for 24 h and the synthesized Zr-MOF was washed and dried. The synthesis mechanism of UiO-66 is illustrated in Figure 5b. During the synthesis, the parameters are the temperature, time, pH, and the composition of the reactants (metal: ligand).

### 4.2. Modification of ZIF-8 and UiO-66

#### 4.2.1. Metal nanoparticles in ZIF-8 and UiO-66: doping

MOFs become up-and-coming alternatives thanks to their uniform distribution of active sites and topological structure [92]. To increase the photocatalyst effect, they are loaded with metal nanoparticles. Thus, effective heterogeneous catalysts are created with the high thermal and mechanical stability of the MOFs. Metal nanoparticles can be loaded into ZIF-8 with several methods. The reaction can occur in several steps [93] or in one step [85]. While metal nanoparticles were loaded into ZIF-8, some researchers produced MOF and nanoparticles separately and then added them together [76,94,95]; the others produced doped ZIF-8 in a single process [96]. Thanh et al. [97] achieved a higher photodegradation efficiency using doped ZIF-8.

For the doping of metal on UiO-66, metal nanoparticles are dissolved in deionized water, added to the UiO-66, and the solution is stirred. The mixture is heated, and the solids formed in this process are centrifuged. Precipitates are washed with distilled water or ethanol, and resulting solids are dried in air or under a vacuum. Doped ZIF-8 and UiO-66 photocatalysts and photocatalysis conditions for various aquatic organic pollutants are comprehensively reviewed in Table 3.

#### 4.2.2. Immobilization of ZIF-8 and UiO-66

The combinations of supporting materials to the MOFs can have higher catalytic activity [98]. It is an essential method to immobilize some functional areas and dynamic groups to increase the porous structure where MOF interacts with target pollutants [99]. Many different substances, such as zeolite, silica, graphene, cotton fabrics, can immobilize MOFs. In addition, high photocatalytic activity and stabilization can also be achieved by immobilization [100]. The deposition of semiconductors on the ZIF-8 is highly influential on photocatalytic activity [101]. Coating and encapsulating ZIF-8 by TiO_2_ or CuO shell increase the organic guest molecules in the micropores of ZIF-8 [64,98].

Cotton fabrics (CF) are frequently used for immobilization due to their flexibility, air permeability, and adsorption ability. Lan et al. [101] studied CF supported ZIF-8 composite (CF@ZIF-8), and the photocatalytic degradation was reached 89%. This composite could be used in large-scale applications with low costs [101]. The CF@ZIF-8 composite was prepared by pretreatment and synthesis steps. Pretreatment for CF follows the steps: sonication of cotton fabric in acetone solution, washing with distilled water, and drying. 2-methylimidazole and methanol are mixed with an ultrasonic mixture, immersing the pretreated CF in the solution, washing with methanol, and drying the solution. The immobilization technique is applied to produce CuO nanoparticles/ZIF-8 composite [98].

For green and cleaner products, Xie et al. [102] produced the organic-inorganic hybrid recyclable catalyst (AILs/HPW/UiO-66-2COOH). These catalysts have both Bronsted and Lewis acid sites, effective for one-pot biodiesel production from low-cost oils.

The acid catalysts were characterized by several techniques, and the results demonstrated that 12-tungstophosphoric heteropolyacid (HPW), a polyoxometalate (POM) acid, was encapsulated. The polyoxometalate-based sulfonated acidic ionic liquids (AILs) were virtually immobilized on the UiO-66-2COOH. Hassabo et al. [103] synthesized a UiO-66-COOH for purified L-Methionine-ɣ-lyase (METase) enzyme from *Wickerhamomyces subpelliculosus*. Subsequently, a new composite (METase@UiO-66) was prepared. The results revealed that greater stability was achieved with the composite as compared to the free enzyme. Moreover, the storage stability of METase was significantly improved after immobilization [103].

### 4.3. Photocatalytic degradation and mechanism

ZIF-8 and its derivatives are commonly used to degrade organic dyes and pharmaceuticals. The degradation of organic aquatic pollutant studies is generally about organic dyes such as methylene blue (MB) and rhodamine B (RhB). Moreover, there are successful studies for methyl orange, rhodamine 6G, Congo red, Reactive Black KN-B, levofloxacin, diazinon, acetaminophen, etc. ZnO-doped on ZIF-8 photocatalysts has been studied by Ökte et al. [76], Liu et al. [104], and Yu et al. [95] for degradation of MB. Yu et al. [95] reported the maximum MB degradation efficiency of 94.1% after 240 min. TiO_2_-doped ZIF-8 photocatalysts for degradation of MB dye were investigated by different researchers [76,84,94]. Chandra and Nath [94] obtained successful results with 93.4% MB degradation in 120 min. The Ag nanoparticles (AgNPs)-doped ZIF-8 photocatalysts for MB degradation were also studied by numerous researchers [104,105]. Chandra and Nath [105] achieved 97.25% MB degradation under UV-visible light at pH 7.89 after 120 min. They produced the most efficient and stable catalyst by using a 300 μL suspension of Ag nanoparticles. When the suspension amount increased, lower degradation efficiency and stability were observed. The charge was transferred from HOMO to LUMO in ZIF-8, so electrons effortlessly passed from the valence band (VB) to the conduction band (CB). Moreover, AgNPs hold the electrons; thus, photo-generated electron transfer became easier in ZIF-8. e- and h+ could react with oxygenated water under the influence of light. Therefore, some of the necessary reactive oxidative species (H+, OH·) were produced for MB degradation.

**Figure 6:**
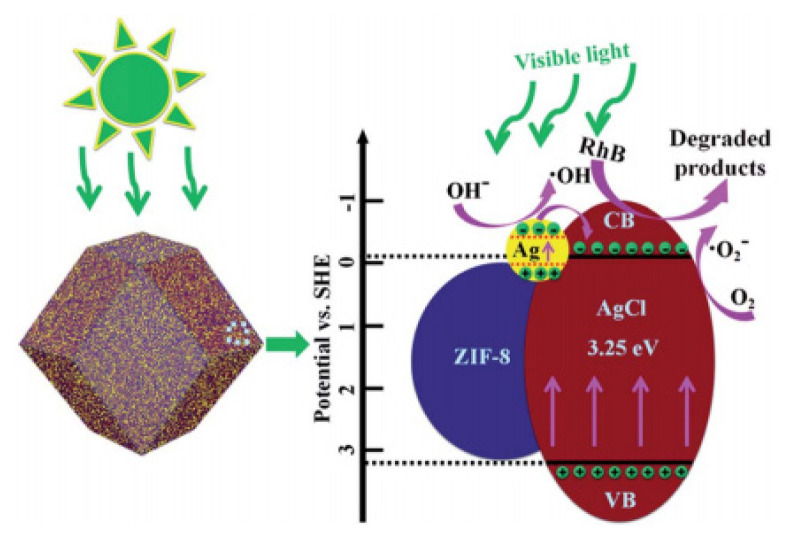
Rhodamine B degradation mechanism by Ag/AgCl@ZIF-8 photocatalyst. Reproduced from ref. [106] with permission from the Royal Society of Chemistry.

The photodegradation mechanism of RhB is given in Figure 6. TiO_2_-doped ZIF-8 was synthesized for degradation of RhB [84,94]. Moreover, Jing et al. [106] and Zuo et al. [107] studied the performances of Ag/AgCl@ZIF-8 for RhB degradation. Among these studies, Jing et al. [106] succeeded with a remarkable 99.12% RhB degradation in 60 min with high stability. The reusability study of Ag/AgCl@ZIF-8 indicated that the photocatalyst was stable and reusable. Visible light could not affect the AgCl molecule directly due to its large band gaps. However, AgNPs could absorb visible light owing to their surface plasmon resonance properties. AgNPs were transferred to the conduction band of AgCls. Then, these particles came together with adsorbed O_2_ and ·O_2_^−^ active molecules. The holes observed on the surface were integrated with OH- to obtain ·OH. Therefore, ·O_2_^−^ and ·OH were acquired to degrade RhB dye [106]. The reactions of RhB degradation are given in (Eqs. (12)–(17)).


(12) $$RhB+Ag/AgCl@ZIF-8\to {\left(\text{RhB}+Ag/AgCl@ZIF-8\right)}_{adsorption}$$

(13) $$Ag/AgCl+hv\to e^{-}+h^{+}$$

(14) $$h^{+}+H_{2}O\to •\;OH+H^{+}$$

(15) $$e^{-}+O_{2}\to O_{2}^{-}$$

(16) $$O_{2}^{-}+{\left(\text{RhB}+Ag/AgCl@ZIF-8\right)}_{adsorption}\to degradation\;products$$

(17) $$•\;OH+{\left(\text{RhB}+Ag/AgCl@ZIF-8\right)}_{adsorption}\to degradation\;products$$

The degradation of diazinon [100], acetaminophen (ACT) [86], and levofloxacin [101] as pharmaceuticals were investigated by using ZIF-8 catalysts. Diazinon was degraded 64.1% after 100 min by Fe_3_O_4_-COOH@ZIF-8/Ag/Ag3PO4 Levofloxacin was degraded 87% after 60 min by Ag/AgCl@ZIF-8/g-C3N4 composite. Furthermore, acetaminophen (ACT) degradation was 99% after 90 min by Ag/AgCl@ZIF-8 catalyst. The photocatalytic mechanism has been examined using different scavengers for trapping holes; superoxide radicals and hydroxyl radicals, ammonium oxalate, benzoquinone, and isopropanol were utilized, respectively. The electrons of ZIF-8 cannot be inspired by visible light irradiation; however, it is possible for Ag/AgCl. Thus, when the energy of an incident photon is larger than the Ag/AgCl band gap value, the electrons on the conduction band pass to the valance band by forming h^+^ and e^−^. Ag nanoparticles located on the AgCl surface could behave as a bridge to supply e^−^ to the surface of ZIF-8 by preventing the reunion of electronic hole pairs. ZIF-8 structure leads to a large specific surface area and provides strong adsorption characteristics. The enrichment of acetaminophen to the catalyst’s surface was considerably accelerated by the efficient adsorption process. The adsorbed acetaminophen was being decomposed.

The primary active substance was O_2_^−^· for the photocatalytic degradation of acetaminophen by Ag/AgCl at ZIF-8. Heterostructure had several roles, such as enhancing the separation efficiency, improving the migration rate of electron hole pairs, decreasing the recombination rate, and enhancing the stability of the catalyst [86].

The band structure of UiO-66 is similar to a semiconductor [108]. UiO-66 and its metal-doped or immobilized variations have been used as photocatalysts for the degradation of aquatic organic pollutants, such as pharmaceuticals and dyes in water [75,108].

Wang et al. [74] produced an AgI/UiO-66 photocatalyst with a band gap energy of 2.82 eV, and 100% degradation of sulfamethoxazole was obtained in 15 min by a xenon lamp. Li et al. [7] investigated the photocatalytic degradation of salicylic acid with changing ratios of UiO-66/BiOI catalyst (30:70, 60:40, 90:10) by using a xenon lamp. It was observed that UiO-66/BiOI (60:40 ratio) catalyst reached 90% degradation in 120 min with a band gap energy of 3.11 eV [7].

RhB degradation has been investigated by using different UiO-66–based catalysts [75,109]. The SnO_2_@UiO-66/rGO catalyst achieved 95.5% degradation in 150 min under visible light with 3.3 eV band gap energy [75]. Using the CdS/UiO-66-NH_2_ catalyst with a band gap energy of 2.28 eV, 95% degradation of RhB was obtained at 60 min under a xenon lamp [109]. Feng et al. [110] also analyzed RhB degradation under xenon lamp irradiation with pure and doped UiO-66 catalyst.

**Figure 7:**
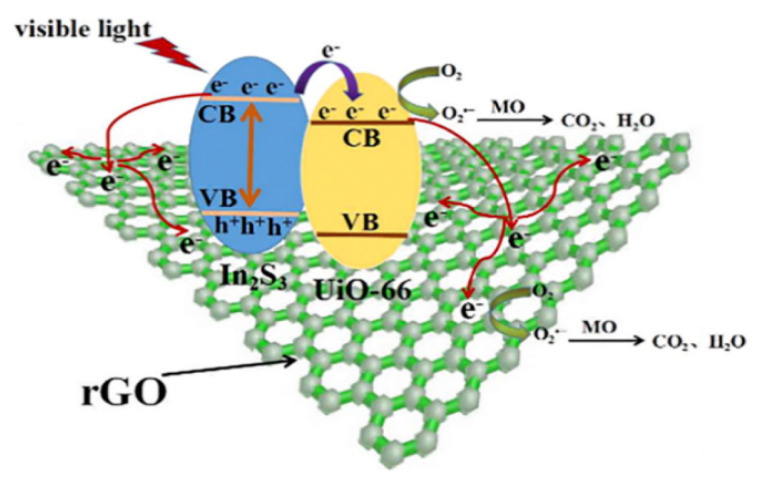
Photocatalysis mechanism of methyl orange with rGO@In_2_S_3_@UiO-66 catalyst [111].

The photodegradation mechanism for methyl orange (MO) via In_2_Sn_3_@UiO-66 was assessed by Gan et al. [111] and 98% degradation was obtained in 60 min. The band gap energy of the catalyst was determined as 2.2 eV [111]. The potential photocatalysis reactions for MO degradation by GO@In_2_S_3_@UiO-66 catalyst under visible light are illustrated in Figure 7 [111].

## 5. Conclusion

The aim of this review article was to focus on the increasing water pollution and treatment of wastewater by using the photocatalytic degradation method. Especially studies on the removal of organic pollutants from wastewater have gained importance recently. Since scientists discovered the role of photocatalysts on degradation performance, they have been working on modification methods of photocatalysts. These methods include metal ion doping, immobilization (on the support surface), morphology control, and semiconductor heterojunction. In the studies, it has been seen that the surface area of the semiconductor is significant, and the increase in the surface area causes an increase in the decomposition efficiency. In this sense, metal-organic frameworks like photocatalysts can be more advantageous in larger internal surface areas and easily tunable organic units. In this study, ZIF-8 and UiO-66 photocatalysts were investigated in detail. The synthesis, modification, and photodegradation mechanisms of ZIF-8 and UiO-66 for organic aquatic pollutants were summarized. Using these photocatalysts, many organic dyes, various pharmaceuticals, and pesticides have been degraded almost completely. According to the researchers, these two photocatalysts are promising for the degradation of aquatic organic pollutants and can be widely applicable in the future, especially with some essential modifications.

## 6. Future prospects

In this study, it is observed that ZIF-8 and UiO-66 are efficient photocatalysts for the degradation of aquatic organic pollutants. By using modification methods such as doping and immobilization, more stable (e.g., in water) and more active photocatalysts with larger surface areas were obtained. At the same time, the band gap can be decreased, which helps to work under visible light [17]. Some issues to be developed are listed below:

Metals with high-value electrons (Fe^3+^, Cr^3+^, Al^3+^, etc.) and transition metals (V^4+^, Ti^4+^, Zr^4+^, etc.) have been used to regulate and increase stability. MOFs containing redox-active metals and organic binders should be produced. Pathways should be sought to produce reusable and cost-effective MOFs [72]. MOFs produced in different amounts, and contents should be examined and optimized under different conditions [45].

There are primarily dye-related studies and fewer studies on other organic pollutants. Thus, it is necessary to increase the studies on other organic pollutants. Studies should be initiated to enlarge the experiments and assist in treating wastewater (containing heavy metals, biological residues, toxic substances) in actual environmental conditions [112].

In order to provide a cleaner and healthier environment and energy saving, studies on this subject should be supported and continued rapidly.

## Figures and Tables

**Table 1:** Organic contaminants and their effects on health.

Organic contaminant	Types	Hazards
Agricultural products	Herbicides, pesticides, fertilizers	Nonbiodegradable, central nervous problems, carcinogenesis, liver and kidney problems, infertility and fatal malformation
Dyes	Anionic, cationic, azo	Toxicity, carcinogenesis, high chromaticity
Pharmaceuticals and personal care products	Antibiotics, antidiabetics, analgesics, make-up products, BPA	Toxicity, EDC, puberty, DNA damage, carcinogenic, muscles and convulsion problems
Artificial sweeteners and food additives	Organoarsenics, sweeteners, sodium benzoate, potassium sorbate	Obesity, diabetes, migraine, genotoxicity, lymphoma, chromosomal aberration, allergy, carcinogenesis
Plastic products	Aromatics, aliphatics, oils	Toxicity, carcinogen, EDC, congenital disabilities, immune system suppression, genetic changes

**Table 2:** Maximum concentrations of some of the contaminants in drinking water.

Contaminant	MCLG (mg/L)	MCL or TT (mg/L)
Agricultural contaminants		
Atrazine	0.003	0.003
Chlorobenzene	0.1	0.1
Diquat	0.02	0.02
Endothall	0.1	0.1
Endrin	0.002	0.002
Picloram	0.5	0.5
Alachlor	zero	0.002
Oxamyl (Vydate)	0.2	0.2
Glyphosate	0.7	0.7
Toxaphene	zero	0.003
Dyes		
1,2,4-Trichlorobenzene	0.07	0.07
Pharmaceuticals and personal care products		
Dichloromethane	zero	0.005
Plastics		
Styrene	0.1	0.1
Vinyl chloride	zero	0.002
Toluene	1	1
Ethylbenzene	0.7	0.7
Ethylene dibromide	zero	0.00005

**Table 3:** Doping of metals on ZIF-8 and UiO-66 and their photocatalytic performance.

Organic pollutant	Catalyst	Band gap energies (eV)	Pollutant initial concentration (mg/L)	Catalyst amount (mg)	Result, photocatalysis (degradation %), (time)	Ref.
Acetaminophen	Ag/AgCl@ZIF-8	5.06	0.5–2	10–80	99%, 1.5 h	[82]
Diazonin	Fe_3_O_4_-COOH@ZIF-8/Ag/Ag_3_PO_4_	5.15	10–80	-	63.4%, 55 min	[97]
Congo red	AgNPs@ZIF-8	5.27	1.6	10	100%, 40 min	[104]
Methylene blue	ZnO@ZIF-8	-	10	10	94.1%, 4 h	[92]
Methylene blue	TiO_2_NPs@ZIF-8	3.43	3.19	10	93.4%, 2 h	[91]
Methylene blue	AgNPs@ZIF-8	5.27	1.6	10	97.25%, 2 h	[104]
Methylene blue	ZnO@ZIF-8	-	2.90	-	11.7%, 100min	[93]
Methylene blue	TiO_2_@ZIF-8	-	2.9	-	16.6%, 100 min	[93]
Methyl orange	ZnO@ZIF-8	-	2.90	-	3.3%, 100 min	[93]
Methyl orange	TiO_2_@ZIF-8		2.90	-	29.1%, 100 min	[93]
Rhodamine B	Ag/AgCl@ZIF-8	5.00	10	50	99.12%, 1 h	[105]
Rhodamine B	Ag/AgCl@ZIF-8	2.85	10	50	99.12%, 1.5 h	[106]
Rhodamine B	TiO_2_@ZIF-8	3.35	2.40	15	61.25%, 2 h	[91]
Rhodamine B	TiO_2_@ZIF-8	3.25	-	-	68.85%, 2 h	[103]
Salycilic acid	UiO-66/BiOI (30:70)	2.45	10	100	80%, 2 h	[7]
Salycilic acid	UiO-66/BiOI (60:40)	3.11	10	100	90%, 2 h	[7]
Salycilic acid	UiO-66/BiOI (90:10)	3.78	10	100	81%, 2 h	[7]
Sulfamethoxazole	AgI/UiO-66	2.82	5	250	100%, 15 min	[108]
Methyl orange	In_2_S_3_@UiO-66	2.2	15	30	98%, 1 h	[112]
Rhodamine B	UiO-66/C_3_N_4_/Ag	2.54	20	20	90%, 3 h	[111]
Rhodamine B	UiO-66	3.77	20	20	50%, 3 h	[111]
Rhodamine B	UiO-66/C_3_N_4_	2.53	20	20	90%, 3 h	[111]
Rhodamine B	UiO-66/Ag	3.54	20	20	60%, 3 h	[111]
Rhodamine B	CdS/UiO-66-NH_2_	2.28	10	20	95%, 1 h	[109]
Rhodamine B	SnO_2_@UiO-66/rGO	3.3	15	50	95.5%, 2.5 h	[110]
